# Role of the Working Alliance on Treatment Outcome in Tailored Internet-Based Cognitive Behavioural Therapy for Anxiety Disorders: Randomized Controlled Pilot Trial

**DOI:** 10.2196/resprot.2292

**Published:** 2013-01-18

**Authors:** Lise Bergman Nordgren, Per Carlbring, Emma Linna, Gerhard Andersson

**Affiliations:** ^1^Department of Behavioural Sciences and LearningLinköping UniversityLinköpingSweden; ^2^Department of PsychologyStockholm UniversityStockholmSweden; ^3^Cerebprivate practiceStockholmSweden; ^4^Swedish Institute for Disability ResearchLinköping UniversityLinköpingSweden; ^5^Department of Clinical Neuroscience,Psychiatry SectionKarolinska InstitutetStockholmSweden

**Keywords:** tailored Internet-based cognitive behavioural therapy, anxiety disorders, working alliance, prediction

## Abstract

**Background:**

Internet-based cognitive behavioral therapy (ICBT) is a form of guided self-help that has been found to be effective for addressing several problems. The target for this type of therapy is usually restricted to one specific disorder. Tailoring the treatment widens the scope of ICBT in that it can address comorbid conditions directly.

**Objectives:**

The working, or therapeutic, alliance has been found to predict outcome in studies of face-to-face therapy. The extent to which these findings apply to ICBT is largely unknown. We therefore decided to find out whether the working alliance could predict outcome in tailored ICBT for anxiety disorders.

**Methods:**

Data were obtained from the treatment group (n=27) in a randomized controlled trial aiming to test the effects of tailored ICBT for anxiety disorders. The forthcoming study was designed to test the hypothesis that the working alliance measured both pre-treatment and early in treatment (week 3) can predict treatment outcome as measured by the Clinical Outcomes in Routine Evaluation–Outcome Measure (CORE-OM) in a heterogeneous group of patients with anxiety disorders (n=27).

**Results:**

Working alliance measured at week 3 into the treatment correlated significantly with the residual gain scores on the primary outcome measure (*r*=-.47, *P*=.019, n=25), while expected working alliance pre-treatment did not (*r*=-.17, *P*=.42, n=27).

**Conclusions:**

These results raise questions about the importance of working alliance in ICBT treatments, and suggest that the working alliance could be important in ICBT.

## Introduction

The working, or therapeutic, alliance is one of the concepts regarded as common to all forms of psychotherapy. Bordin proposed a model consisting of 3 parts—task, bond, and goal—which he stated could apply to all change situations [1]. He suggested that the working alliance was a key factor in these change situations, in which a person tries to achieve change with the assistance of another person, and argued that the concept was universally applicable to all forms of psychotherapy. Following the publication of Bordin’s original paper, the working alliance has been investigated in numerous studies. The results of this research on the relationship between working alliance and outcome indicate that there is a correlation between client-rated working alliance and post-treatment outcome in a variety of disorders and treatments [2,3]. There is evidence for the superiority of client-rated, compared to observer- or therapist-rated, working alliance at the beginning of treatment in predicting outcome [2], however the results are mixed [3,4]. These correlation effects seem to be fairly robust, independent of study design [3] and type of psychological treatment [4], and are replicated in physical rehabilitation [5]. Taken together, there is a body of evidence indicating that working alliance, especially if rated by the client, is an important factor in predicting treatment outcome.

Internet-based cognitive behavior therapy (ICBT) in the form of guided self-help has been proven to be effective for a range of conditions, with effect sizes that equal face-to-face cognitive behavioral therapy [6-10]. There is evidence to suggest that the benefits of ICBT are enhanced by personal contact [11-13]. There is further evidence to suggest that a relationship formed on the Internet is as strong and as deep as its offline counterparts [14]. In a recent review of the therapeutic relationship in the broader field of e-therapy [15], the authors concluded that even though this topic has been investigated, the studies that did take this into account showed that therapeutic relationships or alliance is equivalent to face-to-face therapy, and that there is a relationship between alliance and outcome. Previous studies showed similarities between working alliance measured during face-to-face treatment and working alliance measured during Internet treatment [16-18], by using comparision groups [16,18], and by comparing with earlier research on face-to-face therapy [16]. These findings lead the authors of these papers to conclude that a therapeutic relationship can be formed during Internet-based treatment.

While there is growing evidence to indicate that a working alliance is formed in ICBT [19], not much is known about the possible benefits this may have on outcome in these treatments; this has been investigated with mixed results. For example, Knaevelsrud and Maercker [20] found a nonsignificant correlation between alliance ratings and treatment outcome in patients with post-traumatic stress disorder (PTSD), while a small correlation has been shown for adolescents with anxiety disorders [21].

In a recently published study by our research group [22], no significant correlations were found in the analysis of working alliance as a predictor in patients with generalized anxiety disorder (GAD), social anxiety disorder (SAD), and depression treated with ICBT in 3 separate studies. There are several differences between that study and the one reported here. The participants in the Andersson et al [22] study were included only if their primary diagnosis were matched with the focus of the study (ie, major depression, social anxiety disorder, or generalized anxiety disorder), comorbidities were not assessed or addressed in the treatment, and the ICBT treatment was strictly manualized. In the study presented in this article, we were using the novel format of a tailored ICBT treatment protocol. This may seem like a small difference, but this format makes it possible to address participants’ comorbidities directly, and also to tailor the treatment due to patients’ preferences. We believe that this format is more similar to face-to-face CBT where a personalized functional analysis or case conceptualization is used, and therefore the therapeutic alliance may be shown to yield a stronger correlation with the outcome.

There is also the possibility that, in Internet-based treatments as well in other treatment formats, alliance is at least partly based on patient expectations before treatment and may therefore exist even before the start of therapy [16,23]. The definition of expectation here relates both to the patients’ treatment expectation (eg, beliefs about what the respective roles might be, the treatment format and the duration of treatment), and outcome expectations, which answers questions about the possible beneficial effects of the treatment [24]. We believe that the persons who were recruited to this treatment study knew about the format and length, since this information was clarified on the study website before patients started to fill in the application form, but could have different expectations regarding roles and to what extent this might be a proper treatment for their unique set of problems. The possible differences regarding roles and possible benefits of treatment may stem from the novelty of the treatment format and the fact that neither of the participants had any earlier experience from ICBT.

Little research has been conducted on the predictors of ICBT outcomes. As the use of ICBT broadens, it is increasingly important to establish which factors contribute to outcome. Therefore, the aim of this study was to investigate whether self-reported working alliance, as measured by the Working Alliance Inventory Short form (WAI-S) scale early in treatment (week 3), could serve as a predictor of outcome in tailored ICBT for a heterogeneous group of mixed anxiety disorders. In order to control the possibility of expected working alliance, we also measured alliance before treatment. We expected alliance ratings to be high on the basis of previous research, and measured early in treatment to be a possible predictor of treatment outcome. The potential implication from this study is twofold: (1) to further add to the body of evidence showing that a working alliance can be formed over the Internet and that can equal the alliance rated in face-to-face therapies, (2) if alliance is shown to predict treatment outcome, it could be important for the therapists guiding these programs to work explicitly on trying to foster a working alliance in order to improve outcome.

## Methods

### Recruitment

The data for this study was collected in association with a randomized controlled study of tailored ICBT for anxiety disorders [25], and has not been presented previously. Participants were recruited via newspaper articles in the national and regional press, national radio, interviews about previous studies, and information on a variety of websites. Neither comorbidity nor concurrent medications were criteria for exclusion, provided that anxiety disorder was the principal diagnosis and, if medication was used, that doses were stable for 3 months prior to and during the treatment. Participants were randomized using an online true random number service, independent of the researchers and therapists, to either 10 weeks of individually-tailored, guided Internet-based treatment of anxiety, or to an active control group (online discussion forum). The study was approved by the regional ethical board, and written informed consent was collected from all participants.

### Procedure

Participants applied to participate via the study website and completed an online screening questionnaire in which the CORE-OM [26] served as the primary outcome measure. As part of the screening, participants also completed the WAI-S [27,28] in a slightly modified version, measuring to what extent participants thought that their online therapist would share their point of view, goals, and focus of treatment. This was done before any personal contact with the participant was made. The alliance measure was also completed during the third week of treatment, and post-treatment. Before inclusion, applicants were diagnosed in a face-to-face structured psychiatric interview, Structured Clinical Interview for DSM-IV Axis I disorders (SCID-I) [29]. With exception of the diagnostic interview, all data were obtained using Internet-based questionnaires.

The timing of the WAI-S measurement at week 3 was set in order to measure the alliance before symptom change, where the alliance could be viewed as an indicator of successful treatment [3], but after enough time had passed for the patient and therapist to form a mutual agreement [4,30].

### Participants

The mean age of the 27 participants included was 39.3 years (SD 11.2, range 22–63), 67% (18/27) were female, and 44% (12/27) had a history of previous psychological treatment. 30% (8/27) of the participants had a history of previous, but discontinued anxiolytic or antidepressant medication, 30% (8/27) had continuing medication that had been at a stable dosage for at least 3 months, and 40% (11/27) had no history of any psychiatric medication. The majority of the participants (17/27) were married or living together, while 33% (9/27) reported their marital status as single. Almost half of the participants (12/27, 44%) reported completion of university or college education.

### Treatment

The treatment consisted of a combination of text modules (ie, chapters) previously used in ICBT for panic disorder, social phobia, generalized anxiety disorder, and depression. In total there were 16 modules. Participants were prescribed 6-10 modules to work with during the 10-week treatment period. The treatment, presented in greater detail in Carlbring et al [25], was tailored to match the clients’ unique characteristics and comorbidities (including preferences). Each module included information and exercises, and ended with between 3 and 8 essay style questions, which served as the participants’ homework assignment together with worksheets and reports on the outcome of exercises. The participants were given feedback on their homework assignment from an identified (by name and picture on the study website) therapist, usually within 24 hours. The therapists were 3 master’s students who had completed their clinical training and they were randomly assigned to 7-10 participants each. The therapists were instructed not to exceed 15 minutes/participant/week for email feedback.

### Measures

The primary outcome measure for this study was the CORE-OM [26], a 34-item scale covering 4 subscales. The subscales are: subjective well-being, symptoms (anxiety, depression, physical problems, and trauma), functioning (general functioning, close relationships, and social relationships), and risk (to self and others). Items are scored from 0-4 and relate to the preceding week. CORE-OM clinical scores can range from 0-40, with higher scores indicating greater severity. Internal consistency measured by alpha was reported as .94 [31].

We used a modified version of the WAI-S to measure working alliance. This is a 12-item self-reporting process measure of working alliance [27,28]. The short form of WAI used in this study was generated from the original 36-item scale, Working Alliance Inventory [32], and has 3 subscales—task, bond, and goal—and an overall alliance score (ie, the total score). The scale is available in 3 versions, a client version, a therapist version, and an observer version. The short version consists of the items with the highest load on each of the 3 subscales [33]. Each item is rated on a 7-point scale, with higher scores indicating higher alliance. The WAI-S shows good psychometric properties for both client and therapist versions [33], but large intercorrelations between subscales have been found, and the 3-scale model of WAI and WAI-S remains a subject of debate [27,34,35]. The modifications of the scale were minor, consisting of adaptations for the Internet rather than face-to-face delivery (ie, the word “treatment” was changed to “Internet treatment”, and “contact” was changed to “email contact”). In addition, the WAI-S was adapted according to the assessment point, where the first assessment related to expected alliance. The adaption consisted of changing past and present to future tense. It was also made clear that filling out their expected relationship with their therapist would not affect their possible future participation in the study. We only collected measurements of the client-rated working alliance. Psychometric properties were reported to be excellent for the online version of the WAI-S in Andersson et al [22].

### Treatment Outcome

Results on the CORE-OM showed a large between-group effect size (d=1.00) in favor of the treatment group. The within-group effect size for CORE-OM in the treatment group was d=1.24. A more detailed description of the results is provided in the original report [25].

### Statistical Analysis

We used bivariate correlations and multiple regression models to investigate the possible relationship between outcome and working alliance. For the analysis, we calculated residual gain scores on the composite CORE-OM raw score to control the initial differences pre-treatment, and possible measurement errors due to repeated use of the instrument [36]. Using residual gain scores makes it possible to interpret individual scores relative to typical gains made by other participants at the same initial level [36]. The formula used to calculate residual gain scores was z2 - (z1*r1 2) [36], where z2 represents the z-transformed post-treatment CORE-OM raw score, and *r*1 2 represents the Pearson correlation between pre- and post-treatment assessments.

The residual gain score from CORE-OM served as measurement of treatment outcome, and the client-rated WAI-S score was used as the measurement of working alliance. Changes in alliance were tested by means of paired *t* tests. All statistical analyzes were made with IBM SPSS statistics 19 for Windows.

## Results

Mean alliance ratings on the WAI-S including correlations with residualized gain scores on CORE-OM are presented in Table 1. Alliance ratings increased from pre-treatment to mid-treatment, (*t*_26_=5.5, *P*<.001) and the expected alliance correlated significantly with the early alliance rating (*r*=.57, *P*=.002, n=27). The post-treatment score on the WAI (mean 6.2, SD 0.90) did not change from mid-treatment.

WAI-S measured early in treatment correlated significantly with residualized gain scores from CORE-OM post treatment (*r=-.*47, *P*=.019, n=25), while WAI-S measurements at pre-treatment did not (*r*=-.17, *P*=.425, n=25). Alliance ratings at post-treatment were also correlated with residualized gain scores (*r*=-.42, *P*=.037, n=25).

**Table 1:** Mean working alliance inventory short version ratings pre-, during, and post-treatment, and correlations with residualized change scores on CORE-OM.

	n	Mean (SD)	*r*	*P*
WAI-S total, pre-treatment	27	5.25 (0.72)	-.17	.425
WAI-S total, week 3	25	6.00 (0.80)	-.47	.019
WAI-S total, post-treatment	25	6.20 (0.90)	-.42	.037

When controlling expected working alliance measured pre-treatment, in a multiple regression model the role of the alliance rating during treatment did not change (B=-.53, *P*=.027, n=25) In addition, controlling a history of psychological treatment (B=-.42, *P*=.039, n=25), and levels of symptoms (B=-.44, *P*=.036, n=25) also did not change the association. Controlling age did not change the role of the working alliance (B=-.49, *P*=.016, n=25), whereas controlling gender made the association between working alliance and outcome fall slightly (B=-.40, *P*=.058, n=25).

## Discusion

The aim of this paper was to investigate whether client-rated working alliance could predict outcome, measured by CORE-OM, for a tailored ICBT for anxiety disorders. We found significant correlations between alliance ratings made early in treatment (week 3) and treatment outcome.

ICBT has been found to be effective for a range of psychiatric conditions [18,37], but the underlying mechanisms of change are not well known [38]. There is evidence to indicate better treatment outcome when personal support is combined with online material, compared to unguided self-help [12]. There is, however, also evidence indicating that a technician can be as effective as a therapist [39-41]. Working alliance is regarded as important in face-to-face treatments with a robust, albeit moderate, effect on outcome [4], and as alliance ratings have been shown to be as high in ICBT as in face-to-face therapy [17,18], investigating the possible effect on treatment outcome in this therapy format was of interest. In our sample, we found alliance ratings equal to those typically found in face-to-face therapy, and the tentative conclusion drawn from this study is that it may be possible, at least in a self-recruited sample, to form a working alliance in ICBT. Moreover, alliance ratings increased from baseline, which suggests that ICBT does not lead to worsened alliance. We also found significant correlations between alliance ratings made early in treatment (week 3) and treatment outcome, raising the possibility that early working alliance does affect outcome and might be an important factor even in this treatment format. This is consistent with previous research suggesting that early alliance may predict lower levels of anxiety in cognitive therapy [42]. The findings are, however, mixed in ICBT with regards to the strength of the association between alliance and outcome [20,22]. In this study, we found a significant correlation between working alliance measured early in treatment and outcome in a small sample of patients with comorbidities, and with one third using medications for their condition. If this is explained due to the treatment format of tailoring the treatment, the face-to-face diagnostic interview, or other sample specific variables is not known. These might all be possibilities. In the Andersson et al study [22], the ICBT treatments were manualized and this might be an explanation for the lack of significant alliance outcome associations in their study. This could also be seen in the context of the suggestion by Andrusyna et al [27] that working alliance should be measured differently in CBT than in other forms of psychotherapy. They proposed a 2-factor model consisting of agreement/confidence and relationship. The mixed results may stem from the possibility that the working alliance should be measured differently in CBT.

There has been some debate (eg, [43]) about whether patient expectation could be a factor contributing to early patient-rated working alliance. In an attempt to clarify this, we measured expected working alliance at the time of the application to participate in the study. These measurements were made before any personal contacts had been made with the participants. It is possible that the self-recruited participants in this study were more positive about the treatment format from the start and about the therapists who provided the treatment, even if the data does not suggest this to be the case.

There are other limitations to be considered. Firstly, the small sample size of 27 participants makes it hard to draw conclusions from this study alone, especially since gender seems to affect the association between working alliance on outcome. In this small sample consisting of only 9 men and 18 women, it is hard to take this matter any further, and we believe that this study needs replication with a larger sample to make it possible to explore this issue further. The results are, however, consistent with findings from research on face-to-face therapy, and research showing that Internet relationships can be as important as face-to-face relationships [44].

Secondly, we used the total score of the WAI-S and did not analyze the subscales, given the high intercorrelation between the 3 subscales. This could be seen as a limitation, as we did not investigate whether some subscales showed higher correlation with post-treatment CORE-OM scores. In order to improve the WAI and WAI-S psychometric properties, a new version called Working Alliance Inventory-Short Revised (WAI-SR, [35]) has been developed. Munder et al [34] have shown that WAI-SR distinguished more clearly between the task and bond aspects of the therapeutic alliance, as defined by Bordin’s 3-factor model, making WAI-SR more reliable for subscale division.

Thirdly, the fact that WAI-S was originally designed for face-to-face treatment could be regarded as a limitation. Minor amendments were made for the study (eg, “treatment” was changed to “Internet treatment”, and “contact” to “email contact”), with which we hoped to adapt the scale to this treatment format. As regards measuring outcomes via the Internet, research has shown that this is as reliable as paper-and-pen administration of self-reported measures [45-47].

Fourthly, measurement of the working alliance at week 3 was not accompanied by symptom measurement, so we were unable to estimate to what extent outcome was due to the working alliance and how much was due to the treatment itself.

In spite of the limitations, this study provides some evidence that the quality of the relationship between patient and therapist can be important in Internet treatment, although the working alliance alone may not entirely account for its effectiveness. Our research group has, as a first step, conducted a randomized controlled trial with a larger sample size (n=100) to test the effectiveness of tailored ICBT treatment for anxiety disorders on a primary care population. The next step might be to replicate this study, with the larger sample size, in order to further investigate the relationship between therapist and patient and its effects on outcome.

## Multimedia Appendix 1

CONSORT Ehealth Checklist V1.6.1 [48].

## Abbreviations

CORE-OM: Clinical Outcomes in Routine Evaluation-Outcome Measure
GAD: generalized anxiety disorder
ICBT: Internet-based cognitive behavior therapy
PTSD: post-traumatic stress disorder
SAD: social anxiety disorder
SCID-I: Structured Clinical Interview for DSM-IV Axis I disorders
WAI-S: Working Alliance Inventory Short form
WAI-SR: Working Alliance Inventory-Short Revised
