# Supplementary material for: Role of the Working Alliance on Treatment Outcome in Tailored Internet-Based Cognitive Behavioural Therapy for Anxiety Disorders: Randomized Controlled Pilot Trial
Source: JMIR Res Protoc. 2013 Jan 18;2(1):e4. doi: 10.2196/resprot.2292 (PMC3628159; doi:10.2196/resprot.2292)
Supplement: Supplementary file 1 [file resprot_v2i1e4_app1.pdf]

CONSORT-EHEALTH Checklist V1.6 Report

Manuscript Number 2292

Date completed 8/25/2011 14:50:29

by Lise Bergman Nordgren

Does the working alliance affect treatment outcome in tailored internet-based cognitive behavioral therapy for anxiety disorders?

TITLE

1a-i) Identify the mode of delivery in the title

"tailored internet-based cognitive behavioral therapy"

1a-ii) Non-web-based components or important co-interventions in title

This is specified in the abstract and in the treatment section, but not in the title since we felt that the mode of delivery were more relevant to describe in the title.

1a-iii) Primary condition or target group in the title

This is specified accordingly: "anxiety disorders" in the title.

ABSTRACT

1b-i) Key features/functionalities/components of the intervention and comparator in the METHODS section of the ABSTRACT

This was not applicable in this study scince the intervention has already been reported in detail elsewhere. In the Background section we specified the therapy as "guided self-help" and explaining tailoring of treatment.

1b-ii) Level of human involvement in the METHODS section of the ABSTRACT

In the abstract this is clarified as "Internet-based cognitive behavioral therapy (ICBT) is a form of guided self-help"

1b-iii) Open vs. closed, web-based (self-assessment) vs. face-to-face assessments in the METHODS section of the ABSTRACT

This is not specified in the abstact but in the Methods section of the manuscript.

1b-iv) RESULTS section in abstract must contain use data

This is adressed accordingly: "Working alliance measured at week three into treatment correlated significantly with residual gain scores on the primary outcome measure (r=-.47, P=.019, n=25), while expected working alliance pre-treatment did not (r= -.17, P=.42, n=27)."

1b-v) CONCLUSIONS/DISCUSSION in abstract for negative trials

This is stated in the abstract as: "expected working alliance pre-treatment did not (r= -.17, P=.42, n=27)." To make clear that our hypothesis regarding expected working alliance did not prove to be right.

INTRODUCTION

2a-i) Problem and the type of system/solution

We present the ICBT format as a stand alone format, "Internet-based cognitive behavior therapy (ICBT) in the form of guided self-help has been proven to be effective for a range of conditions, with effect sizes that equal face-to-face cognitive behavioral therapy [6-10]. There is evidence to suggest that the benefits of ICBT are enhanced by personal contact [11-13].", and the tailoring part briefly "In the study presented in this article, we were using the novel format of a tailored ICBT treatment protocol."... "this format makes it possible to address participants co-morbidities directly, and also to tailor the treatment due to patients preferences". This subitem is sort of mixed with subitem 2a-ii in the introduction.

2a-ii) Scientific background, rationale: What is known about the (type of) system

The main part of the introduction is scientific backround and rationale, presenting manualized ICBT as a effective treatment format for a variety of conditions, and in what way we belive that tailoring the treatment might have an impact on the alliance-outcome relationship. Rationale is also given on therapeutic relationship in e-therapy: "In a recent review of the therapeutic relationship in the broader field of E-therapy [15], the authors concluded that even though this topic has been investigated, the studies that did take this into account showed that therapeutic relationships or alliance is equivalent to face-to-face therapy, and that there is a relationship between alliance and outcome.", and in ICBT.

METHODS

3a) CONSORT

"We expected, on the basis of previous research, alliance ratings to be high, and, measured early in treatment, to be a possible predictor of treatment outcome."

3b-i) Bug fixes, Downtimes, Content Changes

No changes were needed, therefore we did not adress this subitem.

4a-i) Computer / Internet literacy

No computer or internet literacy were stated to take part in the study. But, since it is a self-recruited sample, it is possible that a person who felt insecurity regarding their computer skills might not apply to participate.

4a-ii) Open vs. closed, web-based vs. face-to-face assessments:
Tis is specified: "recruited via newspaper articles in the national and regional press, national radio, interviews about previous studies, and information on a variety of websites.", "online screening questionnaire", and "They were later diagnosed in a face-to-face structured psychiatric interview, SCID-I, [27] before inclusion"
4a-iii) Information giving during recruitment
Participants who were interested in application made their application on the study website were all information about the study and procedure could be found. "The study was approved by the the regional Ethical Board, and written informed consent were collected from all participants." The written consent were part of the online screening. Further, "It was also made clear that filling out their expected relation to their therapist would not affect their possible future participation in the study".
4b-i) Report if outcomes were (self-)assessed through online questionnaires
This is adressed in the manuscript; "Participants completed an online screening questionnaire" and; "Data were obtained using internet-based questionnaires before, during and after treatment, and at pretreatment, by face- to-face diagnostic interview."
4b-ii) Report how institutional affiliations are displayed
This is not in the manuscript, but on the study website it was clear that Linköping University, and the department of behavioral siences and learning was responsible för the study.
5-i) Mention names, credential, affiliations of the developers, sponsors, and owners
The treatment is not presented in detail in this manuscript but the full reference to a study wich does this is presented.Treatment is presented as: "The treatment consisted of a combination of previously used text modules used in ICBT of panic disorder, social phobia, generalized anxiety disorder and depression. In total there were 16 modules (i.e. chapters). Participants were prescribed 6 to 10 modules to work with during the 10-week treatment period.".. "Each module included information and exercises, and ended with between three and eight essay-style questions, which served as the participants' home assignment together with work sheets and reports on the outcome of exercises. The participants were given feedback on their homework assignment from an identified (by name and picture on the study website) therapist, usually within 24 hours. The therapists were three MSc students who had completed their clinical training and they were randomly assigned to 7-10 participants. Therapists were instructed not to exceed 15 minutes/participant/week for e-mail feedback." Procedure of measurements relevant for this study is presented; "online screening questionnaire in which the Clinical Outcomes in Routine Evaluation – Outcome Measure (CORE-OM;[26]) served as the primary outcome measure. They were later diagnosed in a face-to-face structured psychiatric interview, SCID-I, [27] before inclusion. As part of the online screening, participants also completed the Working Alliance Inventory- Short (WAI-S; [28, 29]) in a slightly modified version, measuring the expected alliance using the WAI-S measuring to what extent participants thought that their online therapist would share their point of view, goals and focus of treatment. This was done before any personal contact with the participant was made. The alliance measure was also completed during the third week of treatment and at post treatment for the treatment group"
5-ii) Describe the history/development process
"The treatment consisted of a combination of previously used text modules used in ICBT of panic disorder, social phobia, generalized anxiety disorder and depression."
5-iii) Revisions and updating
This is not adressed since no revisions or updates took place.
5-iv) Quality assurance methods
not applicable
5-v) Ensure replicability by publishing the source code, and/or providing screenshots/screen-capture video, and/or providing flowcharts of the algorithms used
not applicable in this study scince we did not elaborate on the actual treatment
5-vi) Digital preservation
not applicable in this study scince we did not elaborate on the actual treatment
5-vii) Access
This is not adressed in the manuscript but a full reference to the article wich did this is provided.
5-viii) Mode of delivery, features/functionalities/components of the intervention and comparator, and the theoretical framework
This is not adressed in the manuscript but a full reference to the article wich did this is provided.
5-ix) Describe use parameters
This is not elaborated other than "Participants were prescribed 6 to 10 modules to work with during the 10-week treatment period", in the manuscript, but a reference on where to find this information is given.
5-x) Clarify the level of human involvement

This is clarified both regarding the inclusion with face-to-face diagnostic interviews and regarding treatment; "participants were given feedback on their homework assignment from an identified (by name and picture on the study website) therapist, usually within 24 hours." "Therapists were instructed not to exceed 15 minutes/participant/week for e-mail feedback."

5-xi) Report any prompts/reminders used

This is not adressed in the manuscript but a redference on where to find this information is given.

5-xii) Describe any co-interventions (incl. training/support)

This is not adressed since no co-interventions were used.

6a-i) Online questionnaires: describe if they were validated for online use and apply CHERRIES items to describe how the questionnaires were designed/deployed

This is reported for WAI-S; "Psychometric properties were reported to be excellent for the online version of the WAI-S in Andersson et al. [22]." but not for CORE-OM.

6a-ii) Describe whether and how “use” (including intensity of use/dosage) was defined/measured/monitored

This is not adressed in our manuscript . For detailed information we refer to Carlbring et al (2011).

6a-iii) Describe whether, how, and when qualitative feedback from participants was obtained

regarding feedback on homework and sort of day-to-day work this is specified"participants were given feedback on their homework assignment". We have not mentioned in the manuscript that we collected qualitative data in the post treatment measurements.

7a-i) Describe whether and how expected attrition was taken into account when calculating the sample size

Not applicable since this study is done on a already published RCT.

7b) CONSORT

Not applicable since no stopping guidelines were used

8a) CONSORT

This is presented; "Participants were randomized by an online true random-number service, independent of the researchers and therapists"

8b) CONSORT

This is presented; "Participants were randomized by an online true random-number service, independent of the researchers and therapists"

9) CONSORT

This is presented; "Participants were randomized by an online true random-number service, independent of the researchers and therapists"

10) CONSORT

This is presented; "Participants were randomized by an online true random-number service, independent of the researchers and therapists"

11a-i) Specify who was blinded, and who wasn’t

Not applicable since noone was blinded.

11a-ii) Discuss e.g., whether participants knew which intervention was the “intervention of interest” and which one was the “comparator”

Not applicable since we only collected data from the treatment group.

11b) CONSORT

Not applicable, we only collected data from the treatment group.

12a) CONSORT

We did not use any comparision group. But the statistical methods were stated: "We used bivariate correlations and multiple regression models to investigate the possible relation between outcome and working alliance. All statistical analyzes were made with IBM SPSS statistics 19 for Windows. The residual gain score from CORE-OM served as measurement of treatment outcome, and the client rated WAI-S score was used as the measurement of working alliance. Changes in alliance were tested by means of paired t-tests."

12a-i) Imputation techniques to deal with attrition / missing values

Not applicable since we did not use any imputation.

12b) CONSORT

"We used bivariate correlations and multiple regression models to investigate the possible relation between outcome and working alliance. All statistical analyzes were made with IBM SPSS statistics 19 for Windows. The residual gain score from CORE-OM served as measurement of treatment outcome, and the client rated WAI-S score was used as the measurement of working alliance. Changes in alliance were tested by means of paired t-tests."

RESULTS

13a) CONSORT

STated in the manuscript as presented n is done in the results section.

13b) CONSORT

This is not adressed in the manuscript but a reference to the article on the RCT were this group was taken from is named.

13b-i) Attrition diagram

This is not adressed in the manuscript but a reference to the article on the RCT were this group was taken from is named.

14a) CONSORT

This is not adressein the manuscript but a reference to the article on the RCT were this group was taken from is named.

14a-i) Indicate if critical “secular events” fell into the study period

not adressed since this did not happen

14b) CONSORT

Not applicable since this did not happen.

15) CONSORT

No table is presented but participants demographics are presented in text in the participanta subsection.

15-i) Report demographics associated with digital divide issues

This is not adressed in the manuscript since this data not were collected.

16-i) Report multiple “denominators” and provide definitions

We did not use any thresholds. All participants in the group were from the original treatment group.

16-ii) Primary analysis should be intent-to-treat

17a) CONSORT

A brief report on outcome is stated when presenting the treatment. For our analyses the manuscript includes a table showing mean and standard deviation, correlations and number of participants for each analysis.

17a-i) Presentation of process outcomes such as metrics of use and intensity of use

Not applicable

17b) CONSORT

A brief report on outcome is stated when presenting the treatment. For our analyses the manuscript includes a table showing mean and standard deviation, correlations and number of participants for each analysis.

18) CONSORT

no subgroup analyses were done.

18-i) Subgroup analysis of comparing only users

Not applicable all participants in the analysis would be stated as users

19) CONSORT

Not stated in teh manuscript since no harming effects were reported.

19-i) Include privacy breaches, technical problems

Not applicable

19-ii) Include qualitative feedback from participants or observations from staff/researchers

Not applicable

DISCUSSION

20-i) Typical limitations in ehealth trials

There are several limitations presented in nthe manuscript; small sample size, using the WAI-S composite scores only, the use of WAI-S wich is orginally developed for face-to-face use, and not using any measurements on symptoms at week three.

21-i) Generalizability to other populations

"the small sample size of 27 participants makes it hard to draw conclusions from this study alone."

21-ii) Discuss if there were elements in the RCT that would be different in a routine application setting

Not applicable since we did not discuss the intervention per se.

22-i) Restate study questions and summarize the answers suggested by the data, starting with primary outcomes and process outcomes (use)

The aim of this paper was to investigate whether client-rated working alliance could predict outcome, measured by CORE-OM, for a tailored ICBT for anxiety disorders. We found significant correlations between alliance ratings made early in treatment (week 3) and treatment outcome.

22-ii) Highlight unanswered new questions, suggest future research

"the small sample size of 27 participants makes it hard to draw conclusions from this study alone. Especially since gender seems to drop the association for working alliance on outcome. In this small sample consisting of only nine men and 18 women it is hard to take this matter any further, and we believe that this study needs replication, with a larger sample to make it possible to explore this further".

Other information

23) CONSORT

not applicable since this trial was not registered.

24) CONSORT

not apllicable. No trial protocol is avavible.

25) CONSORT

Stated as follows: "This research was sponsored by the Swedish Council for Working Life and Social Research (2008-1145)."

X26-i) Comment on ethics committee approval

"The study was approved by the the regional Ethical Board"

x26-ii) Outline informed consent procedures

"written informed consent were collected from all participants."

X26-iii) Safety and security procedures

This is not adressein the manuscript but a reference to the article on the RCT were this group was taken from is named.

X27-i) State the relation of the study team towards the system being evaluated

None of the authors have reported conflicts of interest.
